# Effects of Cover Crops on Water Use Efficiency in Orchard Systems in the Danjiangkou Catchment, Central China

**DOI:** 10.3390/plants14243729

**Published:** 2025-12-07

**Authors:** Linyang Li, Peng Chen, Xinxin Jing, Chenhao Lyu, Runqin Zhang, Xiaoliang Yuan, Qian Li, Yi Liu, Xiaoquan Zhang, Zhiguo Li

**Affiliations:** 1 Wuhan Botanical Garden, Chinese Academy of Sciences, Wuhan 430074, China; 18438615838@163.com (L.L.); chenpeng@wbgcas.cn (P.C.); jingxinxin@wbgcas.cn (X.J.); lyuchenhao@wbgcas.cn (C.L.); 13125081882@163.com (R.Z.); liuyi@wbgcas.cn (Y.L.); 2 Danjiangkou Wetland Ecosystem Field Scientific Observation and Research Station, Chinese Academy of Sciences & Hubei Province, Wuhan 430074, China; 3 Key Laboratory of Aquatic Botany and Watershed Ecology, Wuhan Botanical Garden, Chinese Academy of Sciences, Wuhan 430074, China; 4 University of Chinese Academy of Sciences, Beijing 100049, China; 5 The Nature Conservancy, Beijing 100600, China; xiaoliang.yuan@tnc.org (X.Y.); qian.li@tnc.org (Q.L.); zxiaoquan@tnc.org (X.Z.)

**Keywords:** orchard water management, evapotranspiration, water use efficiency, cover crops, Danjiangkou Reservoir

## Abstract

Water scarcity strongly limits the establishment and productivity of young orchards. Although cover crops are increasingly adopted to improve soil health, their integrated effects on soil–plant–water interactions under drought remain unclear. Here, a two-year field study evaluated Legume, Gramineae, and Legume-Gramineae mixture covers in relation to soil water dynamics, evapotranspiration (ET), and water use efficiency (WUE). Gramineae cover reduced 0–100 cm soil water storage by 5.99% compared with bare soil, whereas the Legume-Gramineae mixture effectively buffered drought-induced water loss. All cover treatments increased total ET, with the mixture showing the highest (10.31%), indicating that enhanced transpiration compensated for reduced soil evaporation. As a result, WUE improved, particularly during winter and spring when water demand was lower. Stepwise analysis identified rainfall as the primary climatic drivers of ET and WUE. Overall, the Legume-Gramineae mixture offers a promising strategy for improving WUE and mitigating drought stress in water-limited orchards.

## 1. Introduction

As a typical perennial and high-value agricultural system, orchards provide economic, ecological, and social benefits. They not only supply fruits and other economic outputs but also significantly influence regional ecology and socio-economic development. However, orchard systems are highly dependent on natural resources—particularly water—while delivering high yields and efficiency [1]. Compared with grain crops, orchards have longer growth cycles, and their peak water demand often coincides with dry periods, making water management more challenging [2]. Against the backdrop of global climate change and increasing water scarcity, drought has become a major constraint on orchard productivity and sustainable development. Improving water use efficiency (WUE)—defined as the biomass or yield produced per unit of water consumed—is thus considered a central goal in orchard management [3]. Enhancing WUE not only helps maintain or increase yield under limited water supply but also supports agricultural sustainability and optimal water allocation. This is especially relevant in orchard systems, where deep root systems, large canopies, and stable water demand mean that WUE affects not only individual orchard performance but also regional agricultural resilience.

In the orchard water cycle, evapotranspiration (ET) is the main pathway of water loss, comprising soil evaporation and plant transpiration [4,5]. As a key process linking soil, plants, and the atmosphere, ET influences soil moisture dynamics, root-zone water availability, photosynthesis, and fruit development [6]. Under frequent drought conditions, regulating ET through improved water management—reducing unproductive water loss and increasing productive water use—is essential for maintaining stable and efficient orchard production.

In recent years, cover crops have been widely adopted in orchard management. These non-economic crops, planted between tree rows or during fallow periods, help improve soil quality, suppress weeds, reduce erosion, and regulate water cycling [7]. Their canopy reduces direct radiation to the soil, lowering evaporation, while their roots enhance soil structure, infiltration, and water retention. As a result, cover crops can increase water infiltration during the rainy season and conserve soil moisture during dry periods.

However, the effect of cover crops on orchard WUE is not straightforward and involves important trade-offs. While they can conserve water by reducing soil evaporation, their own growth and transpiration may increase total ET, potentially offsetting water-saving benefits [8]. For example, ryegrass can reduce soil evaporation by 21.5–27.6% under certain conditions [9], yet it often raises total orchard ET due to high transpiration rates [10]. Similarly, studies on China’s Loess Plateau show that although vegetation cover reduced dry-season soil evaporation, it significantly increased plant transpiration, worsening soil water deficit [11]. The impact of cover crops also depends on species selection, mowing frequency, and seasonal arrangement. Legumes often improve WUE by enhancing nitrogen availability and deep-water use; grasses can boost soil porosity and surface moisture retention [12,13]. However, poor management—such as unchecked cover crop growth during the rainy season—may lead to excessive water use and competition with trees later on [14]. Optimizing species choice and management is therefore crucial for balancing water conservation and production in orchards [15,16,17].

The Danjiangkou Reservoir, a key water source for the Middle Route of the South-to-North Water Diversion Project, is both an ecologically sensitive water conservation area and an important agricultural zone [18]. In recent years, climate change and human activities have increased the frequency of droughts and water shortages, posing serious challenges to local agriculture. At the same time, expanding orchard acreage and intensification have raised irrigation demands, adding pressure on regional water resources. Improving WUE in orchard systems has thus become essential for sustainable agriculture and ecological security in the region.

Understanding how cover crops regulate orchard ET and WUE will not only benefit orchard management but also support water allocation strategies and ecological protection in water-sensitive areas. This study aims to: (1) analyze variations in soil water dynamics and water balance across soil layers under different cover treatments, and (2) compare aboveground biomass and WUE under different cover crop configurations. The findings will provide a scientific basis for sustainable orchard management in the Danjiangkou Reservoir area and other water-limited regions and contribute to regional coordination of agricultural water use and ecological conservation.

## 2. Results

### 2.1. Variations in Environmental Factors

**Figure 1:**
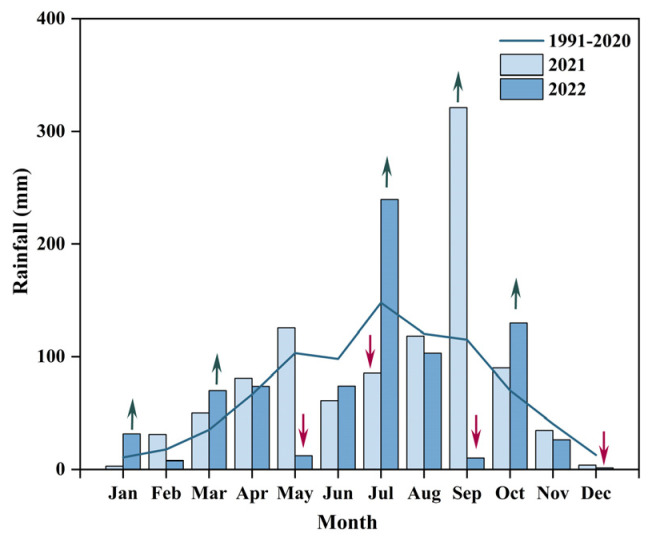
Monthly precipitation during the experimental period compared with the 30-year average precipitation (from TERRACLIMATE, 4 km resolution). Red arrows indicate drought months, while green arrows indicate wetter-than-average months.

Total annual rainfall was approximately 1111.7 mm in 2021 and 779.4 mm in 2022. Compared with the 30-year climatological mean, 2021 was wetter, whereas 2022 was hydrologically normal. Monthly rainfall showed strong fluctuations around the long-term average, with alternating deficits and surpluses throughout the year (Figure 1).

**Figure 2:**
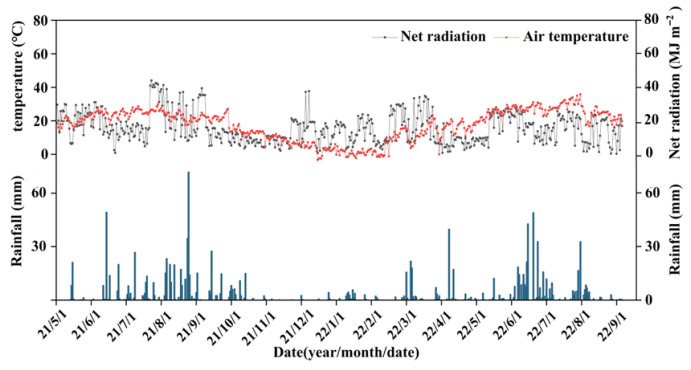
Variation in key meteorological factors during the study period, including precipitation, air temperature, and net radiation. Data were collected from the onsite automatic weather station.

During the experimental period, precipitation was mainly concentrated from June to September (Figure 2), with the maximum daily rainfall of 71.4 mm occurring on 29 August 2021. Air temperature and net radiation exhibited typical seasonal patterns, peaking in summer and declining in winter. These seasonal and interannual climatic variations provided a clear context for assessing how cover crops regulate soil water use under varying hydroclimatic conditions.

### 2.2. Soil Water Content

**Figure 3:**
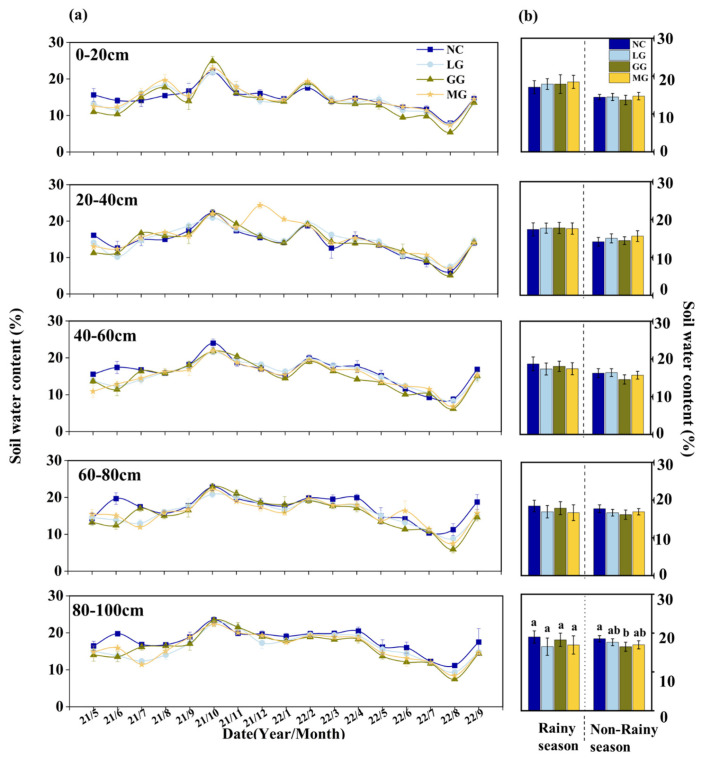
Temporal dynamics of soil water content (SWC) under no cover crop (NC) and three cover-crop treatments: Legume grass (LG, *Medicago sativa*), Gramineae grass (GG, *Festuca arundinacea*), and Mixture Grass (MG, *Medicago sativa* and *Festuca arundinacea*) across the 0–100 cm profile. (**a**) Seasonal SWC dynamics at five soil depths (0–20, 20–40, 40–60, 60–80, and 80–100 cm) during 2021–2022. (**b**) Mean SWC of each treatment in the rainy and non-rainy seasons. Different lowercase letters abouve the bars denote significant differences among treatments at *p* < 0.05 (Tukey’s HSD).

SWC showed similar vertical patterns along the 0–100 cm soil profile across all cover crop treatments (Figure 3a). NC maintained higher SWC at most depths during the normal year, while cover crops increased SWC in the 0–40 cm layer during the wet year but reduced deeper-layer moisture. Seasonally, cover crops generally lowered SWC during the dry period (January–June), except the LG and MG treatments at 20–40 cm, while GG caused the greatest depletion. In the rainy season (July–October), cover crops increased SWC in the 0–40 cm layer but decreased it below 40 cm compared with NC, with LG and MG showing better shallow-layer retention but greater deep-layer depletion.

To further examine how rainfall inputs translated into soil moisture dynamics under different cover crop systems, we analyzed the correlation and lag time between rainfall and SWC (Table 1). Rainfall–SWC correlations were weak or negative across depths, and lag analysis showed delayed responses: up to six months in the 0–40 cm layer under NC and stronger delays in mid-to-deep layers under cover crops, especially LG and MG. These patterns indicate that cover crops altered the temporal coupling between rainfall and soil moisture by enhancing shallow-layer responsiveness while increasing deep-layer buffering.

### 2.3. Soil Water Storage

**Figure 4:**
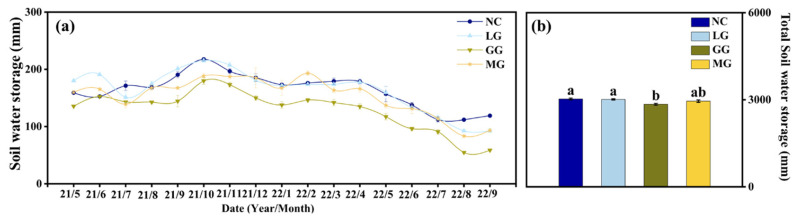
Temporal changes in total soil water storage (**a**) and total water storage (**b**) across the 0–100 cm profile over the entire study period under no cover crop (NC) and three cover-crop treatments: Legume grass (LG, *Medicago sativa*), Gramineae grass (GG, *Festuca arundinacea*), and Mixture Grass (MG, *Medicago sativa* and *Festuca arundinacea*). Different lowercase letters abouve the bars denote significant differences among treatments at *p* < 0.05 (Tukey’s HSD).

Soil water storage (0–100 cm) was higher in autumn and winter but decreased significantly in spring and summer (Figure 4a). During wet periods, LG maintained higher soil water storage following heavy rainfall events (November 2021), whereas under dry conditions (September 2022), MG showed higher water storage than LG despite low rainfall.

The NC treatment (3028.748 mm) showed significantly higher water storage than GG (2847.194 mm, *p* < 0.05), while LG (3012.267 mm) and MG (2951.987 mm) did not differ significantly from NC (Figure 4b). GG consistently showed the lowest overall storage. Soil bulk density showed moderate seasonal variation among treatments (Table 2), and these measured values were used in calculating soil water storage.

### 2.4. Dynamics of ET

**Figure 5:**
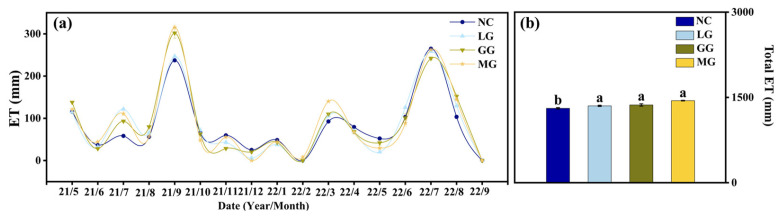
Evapotranspiration (ET) trends for each treatment over time (**a**) and total ET during the entire study (**b**). Error bars indicate standard deviation. Different lowercase letters abouve the bars denote significant differences among treatments at *p* < 0.05 (Tukey’s HSD).

Cover crops led to increased ET relative to NC, indicating greater water use. Cumulative ET was highest for MG (1443.27 mm), then GG and LG (Figure 5b), showing that cover crops—particularly MG. Runoff displayed seasonal differences among treatments (Table 3), and the measured runoff values were incorporated as the R component in estimating actual ET.

### 2.5. Biomass Dynamics of Fruit Trees and Cover Crops

Based on the two-year growth data (Table 4), the shoot growth of fruit trees under different treatments changed dynamically over time. For shoot length, no significant differences were observed among treatments at the initial measurement in September 2021. By the end of the second growing season (September 2022), all treatments showed increased shoot length, though no significant differences were found among them. Although stem diameter measurements showed no statistically significant treatment effects in either year, numerical differences were observed by the second year (MG: 9.57 mm; NC: 11.40 mm).

**Figure 6:**
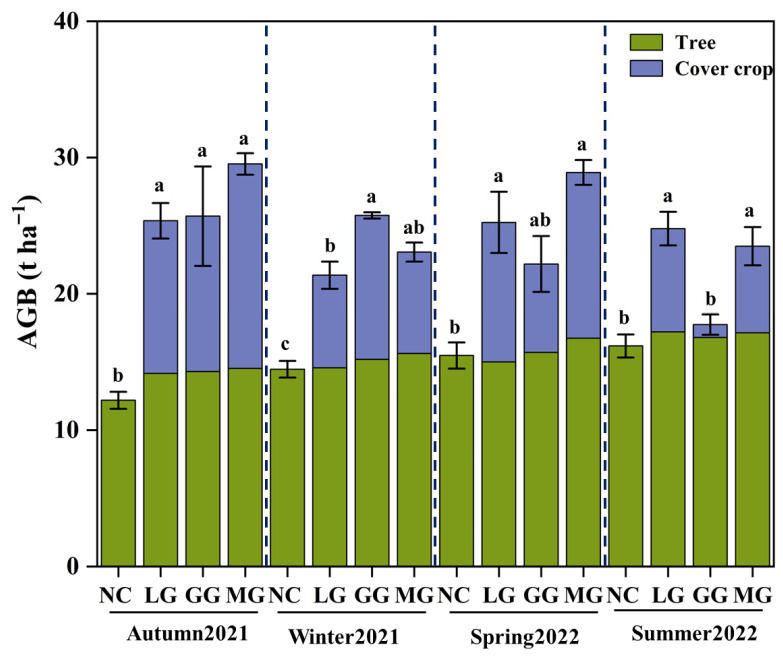
Aboveground biomass (AGB) of fruit trees and cover crop across seasons under different cover crop treatments: no cover crop (NC), and three cover-crop treatments—Legume grass (LG, *Medicago sativa*), Gramineae grass (GG, *Festuca arundinacea*), and Mixture Grass (MG, *Medicago sativa* and *Festuca arundinacea*). Different lowercase letters abouve the bars denote significant differences among treatments at *p* < 0.05 (Tukey’s HSD).

The aboveground biomass (AGB) in this study includes the AGB of both fruit trees and cover crops. Fruit tree growth displayed distinct seasonal dynamics. Biomass accumulation slowed noticeably during the dormant winter period compared to the vigorous growth in spring. Cover treatments generally increased the aboveground biomass of fruit trees (Figure 6), except for the LG treatment in spring (15.48 t ha^−1^), which was slightly lower than NC (15.00 t ha^−1^). The cover crops themselves also showed clear seasonal variation in biomass, performing better in autumn and poorer in summer. Among them, GG had the lowest summer biomass, measuring only 0.94 t ha^−1^.

### 2.6. Seasona Variation in Water Use Efficiency

**Figure 7:**
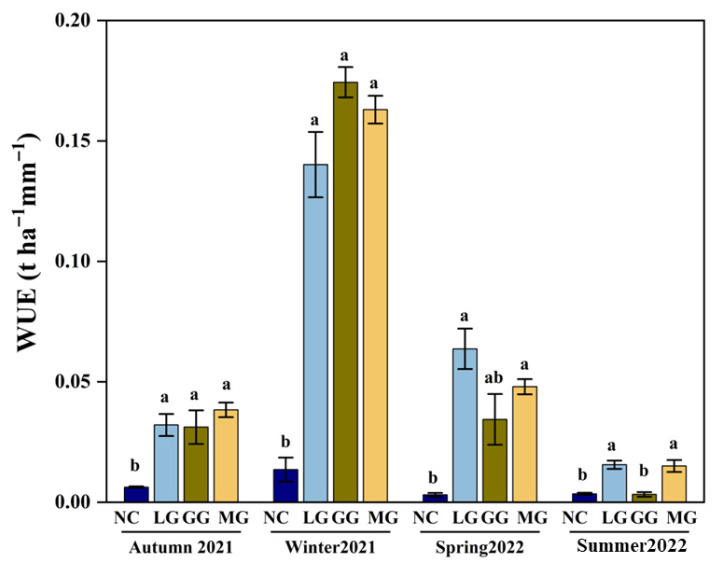
Seasonal water use efficiency (WUE) under different cover crop treatments: no cover crop (NC), and three cover-crop treatments—Legume grass (LG, *Medicago sativa*), Gramineae grass (GG, *Festuca arundinacea*), and Mixture Grass (MG, *Medicago sativa* and *Festuca arundinacea*). WUE was calculated as the ratio of aboveground biomass increment to cumulative evapotranspiration for the entire orchard system. Error bars represent standard deviations. Different lowercase letters abouve the bars denote significant differences among treatments at *p* < 0.05 (Tukey’s HSD).

WUE showed distinct seasonal variation, with generally lower values in summer (0.009 t ha^−1^ mm^−1^) and autumn (0.027 t ha^−1^ mm^−1^), and higher values in winter (0.123 t ha^−1^ mm^−1^) and spring (0.037 t ha^−1^ mm^−1^) (Figure 7). In both spring and autumn, WUE under cover crop treatments was significantly higher than under NC. Among the treatments, MG showed the highest WUE in autumn (0.038 t ha^−1^ mm^−1^), while LG performed best in spring (0.064 t ha^−1^ mm^−1^), indicating season-specific advantages. GG had the lowest WUE in summer (0.003 t ha^−1^ mm^−1^), likely due to high temperature and evapotranspiration, but achieved the highest WUE in winter (0.174 t ha^−1^ mm^−1^), demonstrating strong cold-season performance.

### 2.7. Drivers of SWC, ET, and WUE

Stepwise multiple regression was used to see how environmental factors affect SWC. The factors tested were temperature, radiation, wind speed, humidity, and rainfall. The final model kept only three significant factors: temperature, radiation, and wind speed (Table 5). Together, they explain 37.6% of the variation in SWC (Adjusted R^2^ = 0.38, *p* < 0.001). Temperature was the strongest factor and had a negative effect (β = −0.67, *p* < 0.001). This was followed by the positive effect of radiation (β = 0.14, *p* = 0.001) and the negative effect of wind speed (β = −0.13, *p* = 0.012). Specifically, for every 1 °C increase in temperature, the SWC is predicted to decrease by 0.28 units.

The model of ET (Table 6) kept only four factors: rainfall, wind speed, radiation, and temperature. Humidity was excluded, meaning it did not significantly improve the model after accounting for the other variables. The model explained 72.1% of the variation in ET (Adjusted R^2^ = 0.72, *p* < 0.001). Rainfall was the strongest positive driver of ET (β = 0.87, *p* < 0.001). This shows that water supply is the most important factor in this system. Monthly average wind speed also had a significant positive effect (β = 0.21, *p* = 0.008). This means that over a month, higher wind speed consistently increased ET. Radiation had a negative effect (β = −0.17, *p* = 0.020). The effect of temperature was not significant (β = 0.16, *p* = 0.067).

Model of WUE (Table 7) kept only two significant factors: Humidity and rainfall. It explained 58.1% of the variation in WUE (Adjusted R^2^ = 0.58, *p* < 0.001). Higher humidity significantly improved WUE (β = 0.55, *p* < 0.001), meaning plants used water more efficiently under more humid conditions. In contrast, more rainfall reduced WUE (β = −0.29, *p* = 0.025), suggesting that increased rain lowered water use efficiency. Temperature, radiation, and wind speed were excluded from the model, indicating they did not have a significant direct effect on WUE.

## 3. Discussion

### 3.1. Effects of Cover Crops on Soil Water Dynamics

The soil water responses observed in this study demonstrate that the effects of cover crops are both depth-dependent and species-specific. Although total soil water storage in the 0–100 cm profile decreased significantly only under GG, all cover crops altered the vertical distribution of soil moisture. LG and MG tended to retain more water in the 0–40 cm layer during rainy periods but depleted deeper soil water, whereas GG reduced moisture throughout the entire profile. Such depth-specific patterns are partly consistent with orchard studies in Texas [19] yet differ from reports that alfalfa cover can increase soil moisture under certain conditions [20], highlighting the context-dependent nature of cover crop effects. Stepwise regression identified temperature, radiation, and wind speed as the key factors driving SWC dynamics, this indicates that, after accounting for interactions among multiple factors, thermodynamic and aerodynamic processes had a stronger influence on SWC than direct water input itself. Specifically, temperature had the strongest negative effect, primarily because higher temperatures intensify both soil evaporation and plant transpiration, leading to rapid soil moisture loss. The positive effect of radiation may be because it is often lower on rainy days; its correlation with rainfall may mean it primarily represented low-energy, humid conditions with weaker evaporation in the model. The negative effect of wind speed is linked to its role in enhancing turbulent exchange near the surface and promoting evaporation. Most importantly, rainfall showed no significant independent effect on SWC in the statistical model. This phenomenon is likely due to the water-regulating function of the cover crop system: during rainfall, the plant canopy intercepts and subsequently evaporates part of the rainwater, while the well-developed root systems improve soil permeability, promoting water movement to deeper layers and reducing its retention in the observed zone. In addition, water consumption through transpiration by the cover crops during the growth period may partly offset the replenishing effect of rainfall on soil moisture [21,22,23].

Further analysis showed 5.480% lower soil water storage under GG than under LG (Figure 4b). This contradicted our initial biomass-based hypothesis. The difference likely stems from root architecture and related plant–soil interactions. Legumes like alfalfa develop deep taproots that access deeper water, while Gramineae like tall fescue form dense fibrous roots extracting mainly surface water [24,25]. This allowed LG to use more stable deep-soil moisture, whereas GG intensively depleted surface water. Additionally, legume nitrogen fixation may have indirectly intensified the difference. Fixed nitrogen released into the soil could have stimulated tall fescue transpiration, accelerating surface water use. Together, direct deep-water access and indirect nitrogen effects explain why LG and MG maintained higher soil water levels than GG.

### 3.2. Regulatory Mechanisms of Cover Crops on Evapotranspiration Patterns

Rainfall was the strongest driver of ET in the cover crop system, confirming that water availability is the primary limiting factor for ET in this system. The observed increase in ET under cover crops stems from their efficient use of rainfall and active physiological response. Specifically, the dominant role of rainfall reflects the sensitivity of cover crops to water input. Rainfall not only directly improves soil water availability but also promotes cover crop growth, increasing leaf area index and vegetation cover, thereby enhancing transpiration capacity. During the rainy season, cover crops make full use of effective rainfall and release water back into the atmosphere through vigorous transpiration. During dry periods, their relatively developed root systems can still use soil moisture reserves to maintain a certain level of ET [26,27]. In addition, the positive effect of wind speed shows the accumulated impact of aerodynamic processes at the monthly scale. The negative effect of radiation may be related to soil moisture stress or plant stomatal regulation often associated with high-radiation weather. The non-significant effect of temperature suggests that energy is not a major limiting factor for ET in this system when water is sufficient. However, beyond these environmental drivers, different cover crop types and management practices also play important roles in regulating system ET through distinct biological and physiological pathways.

Different cover types influence ET through distinct pathways: Legume-Gramineae mixture enhance overall system transpiration through niche complementarity [28,29]; Legumes stimulate deep water use efficiency under sufficient water conditions [30]; and Gramineae maintain high evapotranspiration potential through rapid ground cover formation [31]. Timely adjustment of mowing management has been proven to be an effective measure for regulating transpiration water consumption of cover crops [32].

### 3.3. Improvement of Water Use Efficiency by Cover Crops

Cover crops significantly improved orchard water use efficiency, with all treatments showing a consistent seasonal pattern: higher WUE in winter and spring, and lower in summer and autumn [33,34]. This suggests that cover crops did not alter the orchard’s inherent water demand cycle but enhanced the system’s drought resistance by conserving soil moisture, reducing unproductive evaporation, and regulating transpiration.

Stepwise regression showed that only humidity and rainfall significantly affected WUE, together explaining 58.1% of its variation. Higher humidity improved WUE (β = 0.554) because moist air reduces evaporative demand, limiting water loss from plants. In contrast, more rainfall led to lower WUE (β = −0.289). This likely occurs because when water is plentiful, plants focus on rapid growth rather than saving water, which reduces the biomass produced per unit of water used. However, under drier conditions, plants use water more carefully, which increases their efficiency.

WUE in orchard cover crop systems. Differences in WUE among treatments are influenced by their physiological and ecological traits, including drought resistance, morphology, CO_2_ assimilation pathways, and interactions with fruit trees [35]. In this study, Legume-Gramineae mixture covers led to higher WUE than pure Legume covers, especially in winter. This contrasts with previous reports of higher WUE under pure Legume covers [36], a difference that may be due to the inclusion of tall fescue—a C_4_ plant. C_4_ species, with their smaller stomata and rapid response capacity, can enhance WUE under arid or semi-arid conditions [37]. In comparison, Legume cover crops may consume more water for nitrogen fixation, potentially reducing overall WUE. Moreover, the Legume-Gramineae mixture may intensify water competition, leading to slightly lower WUE than pure Gramineae covers [38]. Therefore, cover crops should be selected carefully based on local environmental conditions and water availability to maximize orchard WUE and productivity.

It should be noted that this study used aboveground biomass to estimate WUE. However, in practical orchard management, fruit yield is often of greater concern to farmers. Biomass-based WUE may not fully reflect agronomic benefits; if cover crops increase biomass but reduce fruit yield due to water or nutrient competition, further evaluation using yield-based metrics is necessary [39].

### 3.4. Uncertainty in the Effects of Orchard Cover Crops on Water Indicators

Based on 17 months of observational data, this study provides a preliminary understanding of how cover crops affect water use efficiency in orchards. We recognize that data from a single growing season are limited and cannot fully reflect the long-term impacts of interannual climate variations—particularly rainfall fluctuations—on the orchard water balance. This limitation may also explain why some treatment differences were not statistically significant. Nevertheless, the quantitative results from this young cherry orchard in the Danjiangkou region offer valuable baseline information and practical guidance for local orchard water management under cover crop systems. The observed water regulation patterns and seasonal variations under the normal conditions of 2022 also provide useful insights into how such systems respond to water stress. Even minor treatment effects observed in the short term may still hold agronomic significance under repeated extreme climate events or through long-term accumulation.

In this study, WUE was calculated as the ratio of aboveground biomass to evapotranspiration [40]. Since destructive sampling was not feasible in the productive orchard, aboveground biomass of young cherry trees was estimated using a general allometric model specifically developed for small trees. The model selection was based on the close alignment between the architectural traits of juvenile sweet cherry trees and the growth forms used in the model calibration. Although the model effectively captured relative growth differences across treatments, its generalized nature introduces uncertainty when quantifying the precise effects of cover crops on water use efficiency. Additionally, the relatively low WUE estimates might be related to potential overestimation of ET by the water balance method, which does not fully account for the water allocated to plant growth [41]. Meanwhile, the input and reuse of foliar condensed water may alter the composition of available water sources, further affecting the accuracy of WUE calculations [42]. Future studies should adopt more precise monitoring techniques—such as lysimeters or isotopic mass balance methods—to distinguish between soil evaporation and plant transpiration, thereby improving the reliability of WUE assessment [43].

The short duration of this study limits our ability to fully evaluate the long-term effects of cover crops on orchard water relations. Fruit trees have varying water and nutrient demands at different developmental stages, and tree–cover crop interactions may change fundamentally as the orchard matures. Therefore, establishing a long-term monitoring system is essential to systematically assess how cover crops influence soil moisture, root-zone water competition, and overall water balance across the orchard lifecycle. Long-term data would not only capture the effects of interannual climate variability on soil water and orchard productivity but also help evaluate how management practices—such as mowing timing and frequency—affect cover crop growth and tree water use over time.

Furthermore, differences in root architecture and distribution between fruit trees and cover crops lead to distinct water uptake across soil layers [44]. Under water-limited conditions, this stratified uptake may either intensify or alleviate interspecific competition, ultimately influencing fruit tree growth. In this young cherry orchard, root overlap in shallow soil layers may have triggered water competition between trees and cover crops [45]. However, as tree roots grow deeper and ecological niches differentiate, such competition may gradually diminish. Future research should apply stable water isotope techniques to identify plant water sources, pinpoint critical competition periods.

In summary, while this study reveals the potential of cover crops to regulate orchard WUE within a specific annual context, we fully acknowledge the preliminary and situation-specific nature of these findings. Future work should involve long-term experiments that incorporate multi-annual climate variability and orchard aging dynamics. Such efforts will help verify the comprehensive benefits of cover crops across different hydrological years and management regimes, ultimately supporting more robust and sustainable water management strategies for orchard systems.

### 3.5. Implications for Management Practices

Mowing height and frequency significantly affect WUE and ET. Adjusting these parameters according to weather conditions—both between years and seasons—helps optimize orchard water use [46]. In dry years, higher stubble and less frequent mowing improve residue cover, reducing soil evaporation. During wet periods, lower cutting height and more frequent mowing help control cover crop water use and prevent competition with trees. These adjustments also lower canopy humidity, reducing disease and pest risks.

This study found that cover crops generally increase ET in both wet and normal years, especially from April to June. This period overlaps with fruit maturation and active cover crop growth. We recommend using lower sowing density or selecting less water-demanding species during establishment. Increasing mowing frequency during the growth season can also reduce water competition.

Beyond water relations, management practicality should guide species selection. Alfalfa requires higher initial investment but can reduce long-term nitrogen costs. Tall fescue is easier to manage and needs less frequent cutting. Mixed covers may offer ecological benefits but risk dominance by legumes, increasing management complexity. Future work should integrate cost-benefit analysis and farmer feedback to improve practical adoption.

It should be noted that legume cover crops—typical high water users—are not suitable for large-scale use in dry regions. However, they significantly alter soil water redistribution and evapotranspiration, offering valuable insights into water processes and competition mechanisms. Future studies should examine these water use patterns and, considering both economic costs and farmer input, develop optimized cover crop strategies that support both ecological and economic sustainability in orchards.

## 4. Materials and Methods

### 4.1. Experimental Site and Design

The study was conducted during 2021–2022 in a young cherry (*Prunus avium*) orchard established in 2019 in Xichuan County, Henan Province, China (111°27′24″ E, 33°05′78″ N; 180 m elevation). The orchard features north-south oriented rows with 3.5–4.0 m spacing, with trees not yet in fruiting stage during the experiment.

The experimental design consisted of four soil management treatments arranged in a randomized complete block design with three replications:

NC: No cover crop (bare soil with manual weed removal)
LG: Legume grass (*Medicago sativa*, 12 kg ha^−1^ seeding rate)
GG: Gramineae grass (*Festuca arundinacea*, 18 kg ha^−1^ seeding rate)
MG: Mixture Grass (MG, *Medicago sativa* and *Festuca arundinacea*)

Individual plots measured 80 m^2^, each containing eight trees. Cover crops were maintained at 30–40 cm height by periodic mowing, while NC plots received manual weed control when weeds reached 5 cm height. All plots relied exclusively on natural precipitation.

### 4.2. Site Characteristics and Climate

The experimental site lies in a transitional zone between northern subtropical and warm temperate climates, characterized by temperate continental monsoon conditions. The soil is classified as yellow-brown soil with pH 7.3, bulk density 1.01 g cm^−3^, and contained 15.42 g kg^−1^ organic matter, 0.85 g kg^−1^ total nitrogen, 14.74 g kg^−1^ total carbon, 10.63 mg kg^−1^ available phosphorus, and 85 mg kg^−1^ available potassium.

Meteorological monitoring was conducted using an on-site station equipped with HMP155A temperature/humidity sensors (Vaisala Oyj, Vantaa, Finland), TR-525M tipping-bucket rain gauge, anemometers, pyranometers, and soil temperature/moisture probes at multiple depths (Campbell Scientific, Logan, UT, USA). All data were recorded by a QML201 data acquisition system (Campbell Scientific, Logan, UT, USA). Historical precipitation analysis (1991–2020) utilized TerraClimate data [47], classifying 2021 as a wet year and 2022 as normal. The regional rainy season typically occurs from July to October.

### 4.3. Water Balance Measurements and Calculations

#### 4.3.1. Soil Moisture Monitoring and Storage Calculation

Soil moisture was monitored following a systematic spatiotemporal approach from May 2021 to September 2022. Monthly sampling was conducted at five depth intervals (0–20, 20–40, 40–60, 60–80, and 80–100 cm) within fixed sampling areas established in each treatment plot. At each sampling event, three random soil cores were collected per depth and combined into one composite sample per layer, yielding five composite samples monthly per plot. Soil bulk density was measured quarterly using a 100 cm^3^ cutting ring. This sampling strategy was designed to capture the dynamic nature of soil moisture on a monthly basis while acknowledging the relatively stable character of bulk density, which typically exhibits minimal short-term variation.

All samples were immediately placed in pre-numbered, sealed aluminum boxes upon collection. Fresh weight was measured with an accuracy of 0.01 g to minimize evaporation errors. Samples were then oven-dried at 105 °C until constant weight was achieved (typically 8–12 h), with weight measurements taken every 2 h until consecutive differences were less than 0.002 g. Soil water content (SWC) was determined gravimetrically using:

(1) $$SWC\left(\%\right)=\frac{\left(m_{1}-m_{2}\right)}{\left(m_{2}-m_{0}\right)}\times 100,$$

where *m*_1_ represents the fresh soil weight (g), *m*_2_ represents the dry soil weight (g), and m_0_ represents the weight of the aluminum box (g).

Soil water storage (W, mm) was calculated as:

(2) $$Soil\;water\;storage\left(mm\right)=10\sum_{i=1}^{n}\left(\frac{\theta_{i}}{100}\times \rho \times h_{i}\right),$$

Here, θ_i_ is gravimetric soil water content (%), h_i_ is the soil layer thickness (cm), and ρ is the soil bulk density (g cm^−3^). Change in soil water storage (ΔW) was derived as:

(3) $$\Delta W\left(mm\right)=W_{t2}-W_{t1},$$

where W_t2_ is the soil water storage at time t2 (mm), and W_t1_ is the soil water storage at time t1 (mm).

#### 4.3.2. Evapotranspiration Components

Actual evapotranspiration (ET) was then calculated monthly using the water balance approach [48]:

(4) $$ET\left(mm\right)=P-\Delta W-R-L,$$

where P is the precipitation (mm), ΔW is the change in soil water storage (mm), R is the surface runoff (mm), L is the deep percolation or leaching (mm). Runoff (R) was quantified using galvanized steel sheet enclosures (2 m × 2 m) equipped with collection buckets (Table 4). Leaching (L) was monitored through subsurface collection systems installed at 90 cm depth; however, limited data availability precluded its inclusion in the final ET calculations.

#### 4.3.3. Water Use Efficiency

Seasonal water use efficiency was calculated as:

(5) $$WUE=\frac{A\mathrm{GB}}{ET},$$

where WUE is the water use efficiency (t ha^−1^ mm^−1^), AGB is the aboveground biomass (t ha^−1^), ET is the evapotranspiration (mm).

### 4.4. Biomass Measurements and Calculations

#### 4.4.1. Cover Crop Biomass

Cover crop biomass was sampled seasonally from three randomly placed 1 × 1 m quadrats per plot. Quadrat locations were determined using random coordinates from a fixed central point to ensure unbiased representation. All samples were collected by mowing at the soil surface during each seasonal sampling campaign. All plant material was oven-dried at 65 °C to constant weight for dry mass measurement.

#### 4.4.2. Tree Biomass Estimation

For fruit trees, aboveground biomass was estimated non-destructively using allometric equations. The stem base diameter, measured at 10 cm above ground (D_10_), was determined as the key predictive variable. This measurement was conducted during representative months for each season: September 2021 (autumn), December 2021 (winter), March 2022 (spring), and June 2022 (summer). Shoot length and diameter were measured using a measuring tape and a digital caliper, respectively. The individual tree biomass was calculated using a globally derived allometric model for small trees with the following formula [49]:

(6) $${AGB}_{tree}\left(Kg\right)=e^{\left(2.428\ln D10\right)-3.007}\times 1.128,$$

where AGB_tree_ is the aboveground biomass per tree (kg), D_10_ is the stem diameter at 10 cm height (cm). This method allows for a reasonably accurate estimation of biomass for young fruit trees without causing destructive damage.

### 4.5. Statistical Analysis

Data analysis was performed using Origin 2022 and R 4.3.1. One-way ANOVA was used to test the effects of different treatments on key variables: ET, SWC, AGB, and WUE, with a significance level of α = 0.05. When ANOVA showed significant differences, Tukey’s HSD test was used for post hoc comparisons. To identify the main drivers of these variables, stepwise multiple regression was applied using meteorological factors (temperature, radiation, wind speed, humidity, and rainfall). This method helps avoid misleading results from simple linear regression, which does not account for interactions between variables. We checked all variables for multicollinearity using variance inflation factors (VIF). The regression results include unstandardized coefficients, standardized coefficients (β), and their significance levels to fully assess each factor’s influence.

## 5. Conclusions

This study demonstrates that cover crops exert distinct seasonal and soil-depth-dependent effects on orchard water dynamics. Although cover crops increased total evapotranspiration and reduced water storage in the 0–100 cm soil layer, they significantly improved water use efficiency by enhancing infiltration and reducing runoff. This hydrological regulation creates a more stable “soil water bank,” particularly effective during dry periods and in winter–spring seasons. It is important to note that the effectiveness of cover crops depends on regional climate and soil conditions, requiring appropriate species selection and mowing management to maximize ecological and water conservation benefits. Our findings reveal that cover crops optimize water use by shifting consumption from unproductive losses to ecologically beneficial processes, providing an effective approach for achieving sustainable water management and enhanced ecosystem services in orchard systems.

## Figures and Tables

**Table 1:** Correlation and lag time (months) between soil water content and rainfall under different cover crop treatments.

Depth (cm)	NC		LG		GG		MG	
	Correlation	Lag	Correlation	Lag	Correlation	Lag	Correlation	Lag
0–20	−0.07	6	−0.14	0	−0.18	0	−0.14	0
20–40	−0.12	6	−0.08	6	−0.20	0	−0.31	0
40–60	−0.24	0	−0.22	0	−0.12	0	−0.22	6
60–80	−0.38	0	−0.24	0	−0.25	6	−0.22	0
80–100	−0.30	0	−0.29	6	−0.22	0	−0.20	6

**Table 2:** Variations in soil bulk density under no cover crop (NC) and three cover-crop treatments—Legume grass (LG, *Medicago sativa*), Gramineae grass (GG, *Festuca arundinacea*), and Mixture Grass (MG, *Medicago sativa* and *Festuca arundinacea*).

Time	NC (g cm^−3^)	LG (g cm^−3^)	GG (g cm^−3^)	MG (g cm^−3^)
21.06	1.00 ± 0.23	0.91 ± 0.03	0.98 ± 0.08	1.05 ± 0.08
21.09	1.29 ± 0.07	1.15 ± 0.03	1.11 ± 0.01	1.27 ± 0.03
21.12	1.18 ± 0.01	1.32 ± 0.05	1.17 ± 0.04	1.12 ± 0.03
22.03	1.19 ± 0.08	1.22 ± 0.03	1.13 ± 0.02	1.07 ± 0.09
22.06	1.06 ± 0.06	1.14 ± 0.08	0.90 ± 0.02	1.06 ± 0.05
22.09	1.34 ± 0.05	1.35 ± 0.09	1.30 ± 0.02	1.28 ± 0.05

**Table 3:** Variations in soil runoff loss under different cover crop treatments: no cover crop (NC), and three cover-crop treatments—Legume grass (LG, *Medicago sativa*), Gramineae grass (GG, *Festuca arundinacea*), and Mixture Grass (MG, *Medicago sativa* and *Festuca arundinacea*).

Sampling Time	NC (mm)	LG (mm)	GG (mm)	MG (mm)
21.05	5.74	2.44	2.47	2.29
21.06	22.51	12.32	13.67	10.87
21.07	5.70	2.48	2.43	2.29
21.08	40.60	26.60	26.59	18.58
21.09	11.50	7.16	10.38	7.30
21.10	6.35	2.80	2.32	2.24
21.11	0.60	0.43	0.56	0.13
21.12	0.22	0.08	0.21	0.06
22.01	0.47	0.19	2.00	0.11
22.02	0.38	0.12	0.21	0.10
22.03	4.00	1.49	2.02	1.53
22.04	4.26	1.52	2.71	1.58
22.05	0.22	0.18	0.40	0.17
22.06	4.35	1.49	2.49	2.02
22.07	25.34	16.79	17.42	15.21
22.08	9.24	6.78	7.82	4.79
22.09	0.20	0.10	0.13	0.02

**Table 4:** Effects of different treatments on shoot growth of fruit trees (based on two dynamic measurements).

Treatment	Shoot Length (cm)		Stem Diameter (mm)	
	2021.09	2022.09	2021.09	2022.09
NC	24.88 ± 4.21 a	33.65 ± 1.74 a	6.30 ± 0.39 a	11.40 ± 0.52 a
LG	19.35 ± 4.38 a	34.15 ± 1.47 a	5.28 ± 0.42 a	10.03 ± 0.48 a
GG	19.90 ± 3.07 a	30.92 ± 1.80 a	5.86 ± 0.18 a	10.58 ± 0.66 a
MG	19.38 ± 2.73 a	29.06 ± 2.00 a	5.59 ± 0.36 a	9.57 ± 0.30 a

Note: The letter “a” indicates no significant difference among treatments (*p* < 0.05).NC, no cover crop; LG, Legume grass (*Medicago sativa*); GG, Gramineae grass (*Festuca arundinacea*); MG, Mixture grass (*Medicago sativa* and *Festuca arundinacea*).

**Table 5:** Results of stepwise multiple regression analysis for factors influencing soil water content (SWC).

Variable	Unstandardized Coefficient (B)	Standard Error	Standardized Coefficient (β)	*t*-Value	*p*-Value
(Intercept)	21.09	1.16	-	18.18	<0.001
Temperature (℃)	−0.28	0.02	−0.67	−13.20	<0.001
Radiation (MJ m^−2^)	0.09	0.03	0.14	3.21	0.001
Wind speed (m s^−1^)	−3.41	1.35	−0.13	−2.53	0.012

**Table 6:** Results of stepwise multiple regression analysis for factors influencing evapotranspiration (ET).

Variable	Unstandardized Coefficient (B)	Standard Error	Standardized Coefficient (β)	*t*-Value	*p*-Value
(Intercept)	46.24	38.59	-	−1.20	0.235
Rainfall (mm)	0.87	0.08	0.87	10.82	<0.001
Temperature (°C)	1.43	0.77	0.16	1.86	0.067
Radiation (MJ m^−2^)	−2.32	0.99	−0.17	−2.35	0.020
Wind speed (m s^−1^)	122.85	44.56	0.21	2.76	0.008

**Table 7:** Results of stepwise multiple regression analysis for factors influencing water use efficiency (WUE).

Variable	Unstandardized Coefficient (B)	Standard Error	Standardized Coefficient (β)	*t*-Value	*p*-Value
(Intercept)	−0.20	0.07	-	−2.88	0.006
Humidity (KPa)	0.36	0.08	0.55	4.44	<0.001
Rainfall (mm)	−1.00 × 10^−3^	−1.00 × 10^−3^	−0.29	−2.32	0.025

## Data Availability

The raw data supporting the conclusions of this article will be made available by the authors on request.
